# FOXA1 is a highly sensitive diagnostic marker for prostate cancer including small cell carcinoma of the prostate

**DOI:** 10.1111/his.70166

**Published:** 2026-05-07

**Authors:** Jianping Zhao, Jun Yao, Hossein Hosseini, Ezra Baraban, Ruihe Lin, Charles C. Guo, Lei Huo, Wei Lu, Khaja Khan, Shufang Wang, Luisa Maren Solis Soto, Qingqing Ding

**Affiliations:** ^1^ Department of Anatomical Pathology The University of Texas MD Anderson Cancer Center Houston Texas USA; ^2^ Department of Molecular and Cellular Oncology The University of Texas MD Anderson Cancer Center Houston Texas USA; ^3^ Department of Pathology The Johns Hopkins Hospital Baltimore Maryland USA; ^4^ Department of Translational Molecular Pathology The University of Texas MD Anderson Cancer Center Houston Texas USA

**Keywords:** biomarker, FOXA1, prostate adenocarcinoma, small cell carcinoma

## Abstract

**Aims:**

Prostate cancer is one of the most common malignancies in men and a major cause of cancer‐related mortality worldwide. While second‐generation antiandrogens induce durable responses, they have also led to the emergence of aggressive, androgen‐independent subtypes of prostate cancer, including small cell carcinoma of the prostate. The diagnosis of these subtypes remains challenging due to the frequent loss of traditional prostatic markers, and novel prostate‐specific markers are needed in routine pathology practice.

**Methods and results:**

Through analysis of the Cancer Genome Atlas (TCGA) database, we identified Forkhead box protein A1 (FOXA1) as a potential diagnostic marker for prostatic cancer. We compared FOXA1 and NKX3.1 expression in benign prostatic glands and prostatic adenocarcinoma; FOXA1 immunostaining demonstrated a similar nuclear staining pattern to NKX3.1 and a high sensitivity for prostatic adenocarcinoma. Notably, in small cell carcinoma of the prostate, which typically loses expression of traditional prostate markers, FOXA1 expression was detected in 8 of 10 (80%) primary cases and 12 of 21 (57%) metastatic cases in our cohort. We also evaluated FOXA1 expression across various tumour types and observed positivity in 25 of 44 (57%) breast carcinomas, 15 of 68 (22%) urothelial carcinomas and rarely in other tumour types. Among 106 neuroendocrine tumours/carcinomas from different organs, only 2 of 4 breast neuroendocrine carcinomas showed FOXA1 expression.

**Conclusions:**

FOXA1 is a highly sensitive diagnostic marker for prostate cancer. It can serve as a valuable adjunct for confirming prostatic origin in diagnostically challenging cases such as prostatic small cell carcinoma.

## Graphical abstract

FOXA1 is a highly sensitive diagnostic marker for prostate cancer. Besides prostatic adenocarcinoma, it is also expressed in 57%–80% of metastatic or primary prostatic small cell carcinomas, which lose traditional prostatic markers including NKX3.1. Although it is less specific than NKX3.1, it can serve as a valuable adjunct for confirming prostatic origin in diagnostically challenging cases with loss of traditional prostatic markers.

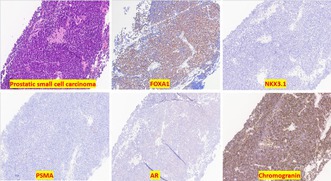

**Abbreviations**
AR: androgen receptor
FFPE: formalin‐fixed paraffin‐embedded
FOXA1: forkhead box protein A1
IHC: immunohistochemical
PSA: prostate‐specific antigen
PSMA: prostate‐specific membrane antigen
TCGA: The Cancer Genome Atlas
TCGA: Through analysis of the Cancer Genome Atlas
TMAs: tissue microarrays

## Introduction

Prostate cancer remains one of the most common malignancies affecting men globally and in the United States. According to the American Cancer Society, 313,780 new cases (30% of all new cancer diagnoses in men) and 35,770 prostate cancer‐related deaths (11% of all cancer deaths in men) are estimated in the United States in 2025.^1^ Standard treatment options for high‐risk prostate cancer include androgen deprivation therapy, radiation and/or radical prostatectomy. The introduction of second‐generation antiandrogens elicits prolonged responses; however, it also leads to the emergence of resistance mechanisms that are independent of the androgen pathway.^2^ The resulting androgen‐independent phenotype is typically associated with an aggressive clinical course and poor prognosis. One of the common aggressive subtypes is small cell carcinoma of the prostate.^3^, ^4^ The pathological diagnosis of prostatic small cell carcinoma is particularly challenging because these tumours often display histological features distinct from conventional prostatic adenocarcinoma and frequently lose expression of traditional prostatic markers such as NKX3.1, androgen receptor (AR), prostate‐specific antigen (PSA), prostate‐specific membrane antigen (PSMA), prostein and ERG. Clinically, this loss of immunophenotypic identity complicates the determination of whether a metastatic tumour represents progression of known prostatic carcinoma or a new primary malignancy derived from another site of origin. These diagnostic difficulties underscore the need for new prostate‐specific markers in daily pathology practice.^5^

To address this need, we analysed data from The Cancer Genome Atlas (TCGA) and identified Forkhead box protein A1 (FOXA1) as a potential marker for prostate cancer. We subsequently evaluated FOXA1 expression in both primary and metastatic prostatic adenocarcinoma, as well as in small cell carcinoma of the prostate. FOXA1 expression was also compared with NKX3.1, a well‐established prostate‐specific marker. In addition, we explored the expression of FOXA1 across a wide spectrum of tumour types, including carcinomas of the breast, bladder, lung, colon, thyroid, kidney, liver as well as testicular germ cell tumours and well‐differentiated neuroendocrine tumours and neuroendocrine carcinomas of multiple organs.

## Materials and methods

### TCGA database analysis

TCGA gene expression data for prostate adenocarcinoma and 30 other tumour types were downloaded from the Broad Institute Genomic Data Analysis Centre website (https://gdac.broadinstitute.org/). To identify potential prostate cancer marker genes, we applied the following filtering criteria: overall median mRNA expression level in prostate cancer is greater than 4 (log_2_ transcripts per million value) and at least twofold higher than median expression in other tissues; median nonzero expression level in prostate cancer is at least 1.5‐fold higher, where zero expression is defined as log_2_ transcripts per million value below −8.8; top 80% percentile lower line for expression in prostate cancer is higher than top 20% percentile lower line in other tumours, allowing at most 2 exceptions.

### Human tumour samples

This study was approved by the Institutional Review Board of The University of Texas MD Anderson Cancer Center and The Johns Hopkins Hospital. Patient consent was received for the patient's tissue used in this study. Whole‐slide formalin‐fixed paraffin‐embedded (FFPE) sections were used for 21 primary prostatic adenocarcinomas, 11 metastatic prostatic adenocarcinomas, 31 prostatic small cell carcinomas and 5 neuroendocrine carcinomas of the bladder. For other tumour tissues, tissue microarrays (TMAs) were used and purchased from TissueArray LLC (Derwood, MD), including 60 prostatic adenocarcinoma (PR633a), 58 urothelial carcinomas (BC12011d), 125 colon adenocarcinomas (CO1251), 70 lung cancers (LC1503), 40 lung small cell cancers (LC10010d), 64 thyroid cancers and adenomas (TH802b), 59 renal tumours (KD1501a), 96 hepatocellular carcinomas (LV1021a), 68 well‐differentiated neuroendocrine tumours of multiple organs (NE842) and 54 testicular germ cell tumours (TE810). In addition, a TMA of 44 invasive breast cancers was made in the Department of Pathology and Translational Molecular Pathology at The University of Texas MD Anderson Cancer Center.

All diagnoses were independently confirmed by two pathologists based on tumour morphology and immune profile if available. The diagnosis of small cell carcinoma of prostate was established using three criteria: (1) tumour shows classic morphology of small cell neuroendocrine carcinoma; (2) tumour is positive for at least one neuroendocrine marker (synaptophysin, chromogranin, INSM1 or CD56); and (3) tumour is negative for all traditional prostatic markers (NKX3.1, PSMA, PSA, AR and Prostein).

### Immunohistochemical analysis

Immunohistochemical (IHC) staining was performed with a mouse monoclonal antibody against human FOXA1 (FOXA1/1514, NeoBiotechnologies) in a Leica Bond Max autostainer system (Leica Biosystems, Nussloch, GmbH) according to standard automated protocols. Briefly, 4‐μm thick formalin‐fixed, paraffin‐embedded tumour tissue sections were deparaffinized and rehydrated according to the Leica Bond protocol. Antigen retrieval was performed, and slides were incubated with FOXA1 antibody (1:1000). The primary antibody was detected using diaminobenzidine chromogen and counterstaining with haematoxylin.

FOXA1 immunostains were independently reviewed by two pathologists, and only nuclear staining was considered positive. Immunoreactivity scores were calculated by multiplying the number representing the percentage of immunoreactive cells (0, <1%; 1, 1%–10%; 2, 11%–50%; 3, 51%–100%) by the number representing staining intensity (0, negative; 1, weak; 2, moderate; 3, strong). The immunoreactivity scores were considered negative (0–1), low positive (2), intermediate positive (3, 4) or high positive (6 and 9).

## Results

### FOXA1 and NKX3.1 mRNA levels in prostate adenocarcinoma and 30 other tumour types

**Figure 1:**
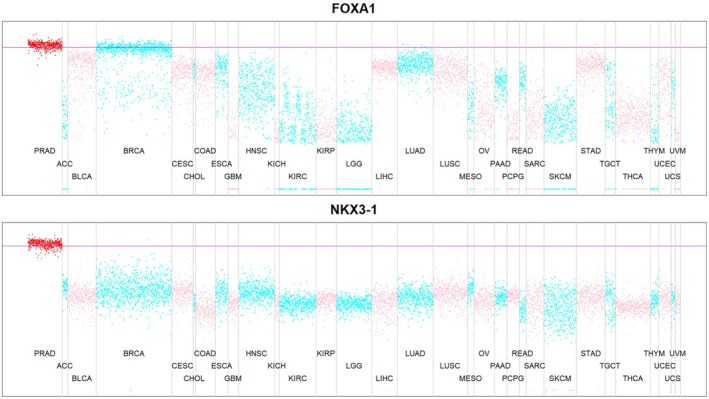
mRNA levels of FOXA1 and NKX3.1 in prostate adenocarcinoma and 30 other tumour types based on TCGA data. Purple line is the top 80% expression in prostate adenocarcinoma (PRAD). Analysed TCGA tumour types include adrenocortical carcinoma (ACC), urothelial carcinoma (BLCA), breast invasive carcinoma (BRCA), cervical squamous cell carcinoma and endocervical adenocarcinoma (CESC), cholangiocarcinoma (CHOL), colon adenocarcinoma (COAD), oesophageal carcinoma (ESCA), glioblastoma (GBM), head–neck squamous cell carcinoma (HNSC), chromophobe renal cell carcinoma (KICH), clear cell renal cell carcinoma (KIRC), renal papillary carcinoma (KIRP), low‐grade glioma (LGG), hepatocellular carcinoma (LIHC), lung adenocarcinoma (LUAD), lung squamous cell carcinoma (LUSC), mesothelioma (MESO), ovarian serous cystadenocarcinoma (OV), pancreatic adenocarcinoma (PAAD), pheochromocytoma (PCPG), prostate adenocarcinoma (PRAD), rectal adenocarcinoma (READ), sarcoma (SARC), skin cutaneous melanoma (SKCM), stomach adenocarcinoma (STAD), testicular germ cell tumour (TCGT), thyroid carcinoma (THCA), thymoma (THYM), uterine corpus endometrial carcinoma (UCEC), uterine carcinosarcoma (UCS) and uveal melanoma (UVM).

To identify new prostate‐specific tumour markers, we systematically analysed mRNA expression data across 31 solid tumour types using the TCGA database. As shown in Figure 1, FOXA1 mRNA levels were notably elevated in prostate adenocarcinoma and breast carcinoma (which is rare in men) compared to other tumour types. This suggests that FOXA1 may serve as a potential biomarker for prostate cancer. In comparison, NKX3.1 expression was significantly elevated only in prostate adenocarcinoma, consistent with its well‐established specificity as a prostatic marker.

### FOXA1 and NKX3.1 expression in benign prostatic glands and prostate adenocarcinoma

**Figure 2:**
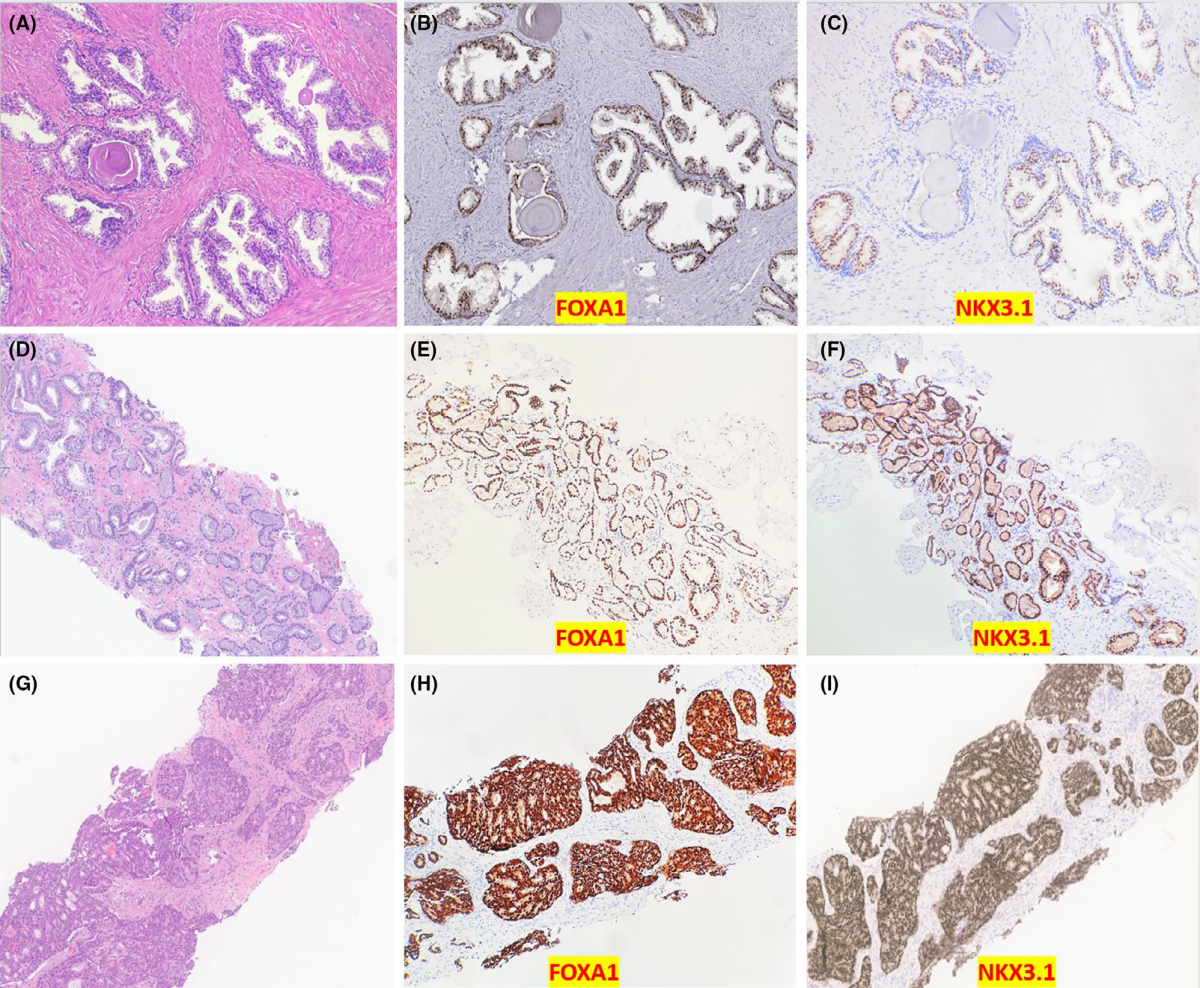
FOXA1 expression in benign prostatic glands and prostatic adenocarcinoma. Similar to NKX3.1, FOXA1 shows diffuse nuclear expression in the luminal cells of benign prostatic glands (**A–C**), low‐grade (**D–F**) and high‐grade (**G–I**) prostatic adenocarcinoma.

We next assessed FOXA1 protein expression in benign prostatic glands and adenocarcinoma (Figure 2). Similar to NKX3.1, FOXA1 showed a diffuse nuclear expression in luminal cells of benign prostatic glands, with no staining in basal cells. Limited immunohistochemical studies in other normal tissues demonstrated that FOXA1 was expressed in nuclei of normal breast epithelium and bladder urothelium. By contrast, FOXA1 was not detected in the seminal vesicle, liver, renal parenchyma, testis, thyroid, lung or gastrointestinal tract tissues (data not shown). In prostatic adenocarcinoma, FOXA1 immunostain demonstrated a similar nuclear staining pattern to NKX3.1 and a high sensitivity with positive staining in 76 of 81 (94%) primary and all 11 metastatic cases as well as 4 of 4 cases of prostatic adenocarcinoma with Paneth cell‐like differentiation (data not shown). Most cases showed intermediate to high FOXA1 positivity (Table 1).

**Table 1:** FOXA1 expression in prostatic adenocarcinoma and small cell carcinoma

FOXA1 expression		Negative	Positive			Total
			Low	Intermediate	High	
Prostatic adenocarcinoma	Primary	5 (6%)	6 (7%)	25 (31%)	45 (56%)	81 (100%)
	Metastatic	0	0	2 (18%)	9 (82%)	11 (100%)
Prostatic small cell carcinoma	Primary	2 (20%)	2 (20%)	2 (20%)	4 (40%)	10 (100%)
	Metastatic	9 (43%)	5 (24%)	4 (19%)	3 (14%)	21 (100%)

### FOXA1 expression in small cell carcinoma of the prostate

It is well known that small cell carcinoma of the prostate frequently loses expression of traditional prostatic markers, including NKX3.1, PSA, PSMA and/or AR. Indeed, loss of these markers, along with neuroendocrine marker expression and classic histology, is commonly used to support the diagnosis. We analysed 10 primary and 21 metastatic prostatic small cell carcinoma cases. Interestingly, despite the loss of traditional prostatic markers, significant FOXA1 expression was detected in 8 (80%) of 10 primary tumours and 12 (57%) of 21 metastatic tumours (Table 1).

**Figure 3:**
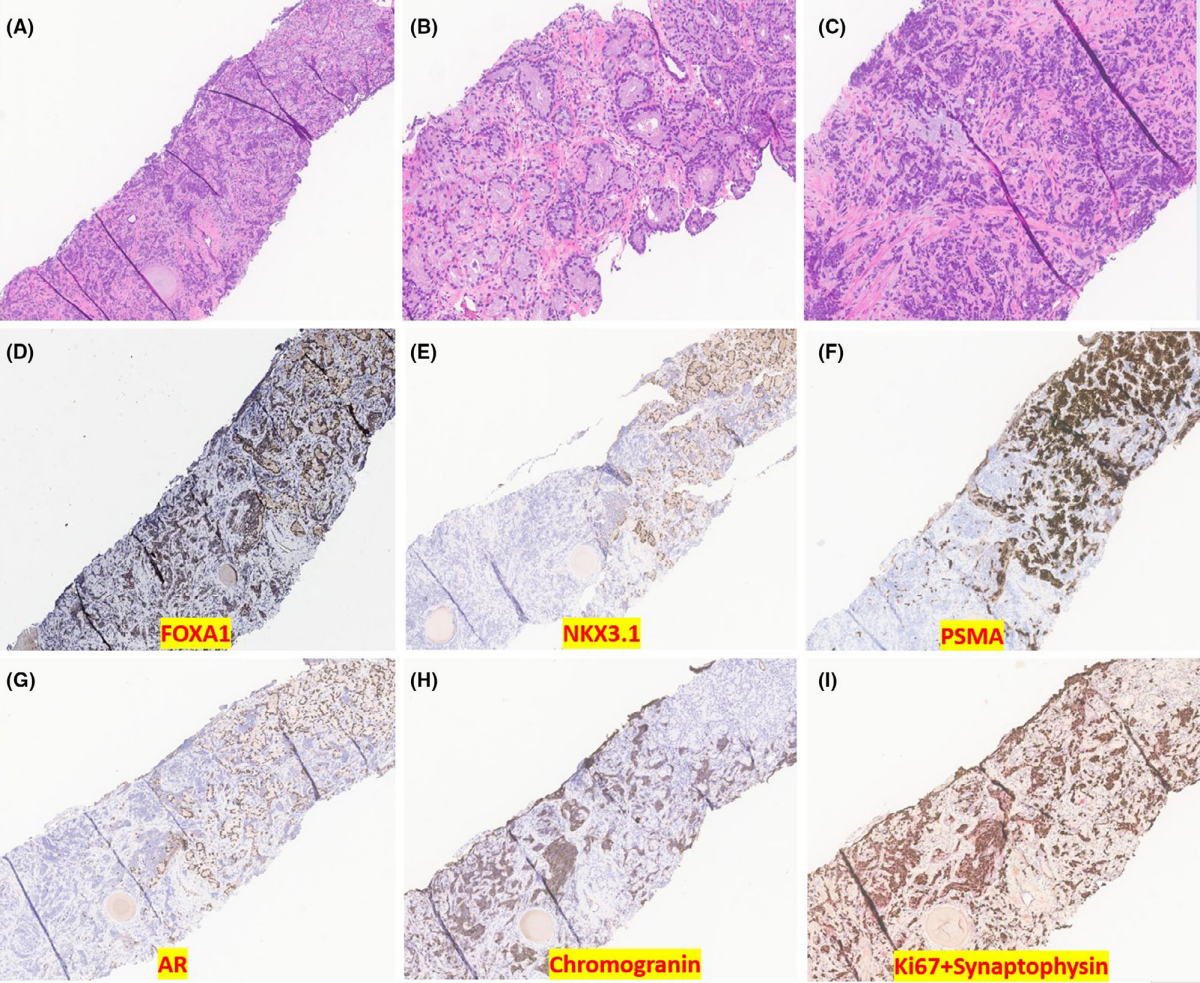
FOXA1 expression in a primary de novo small cell carcinoma of the prostate. (**A–C**) This prostate core biopsy shows mixed prostatic adenocarcinoma (**B**) and small cell carcinoma (**C, D**) Both prostatic adenocarcinoma and small cell carcinoma show diffuse expression of FOXA1; however, the small cell carcinoma component loses expression of traditional prostatic markers including NKX3.1, PSMA, AR (**E–G**), while expressing neuroendocrine markers including synaptophysin and chromogranin with a high Ki67 index (**H, I**). [Colour figure can be viewed at wileyonlinelibrary.com]

**Figure 4:**
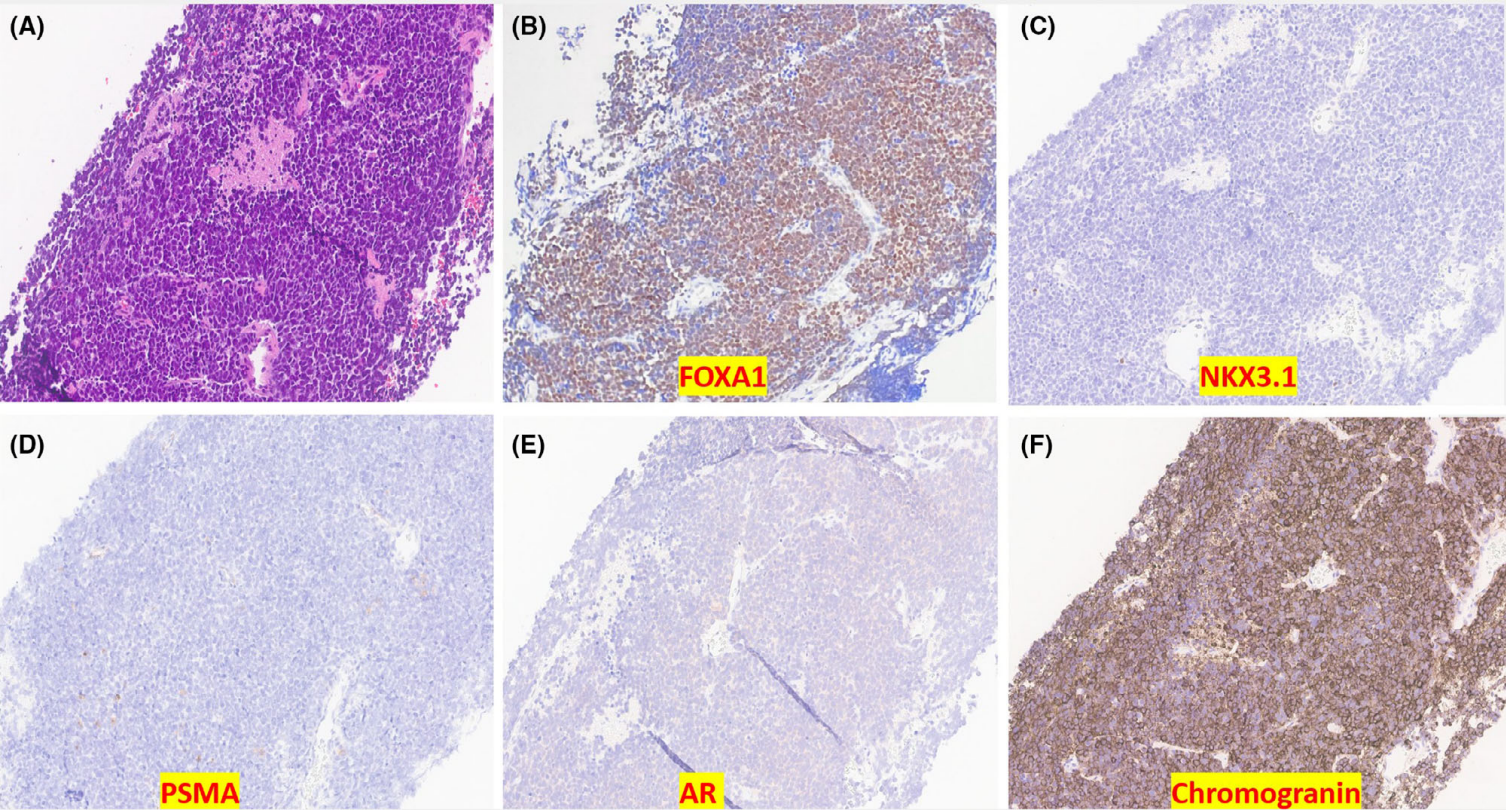
FOXA1 expression in a metastatic small cell carcinoma of the prostate. This pelvic lymph node biopsy was from a 68‐year‐old male with a history of high‐grade prostatic adenocarcinoma status post radiation/hormonal therapy as well as radical prostatectomy. (**A**) Tumour shows histological features consistent with small cell carcinoma, and clinical history was suggestive of prostatic primary; (**B**) FOXA1 is strongly expressed in the tumour cells, whereas other prostatic markers are negative, including NKX3.1, PSMA and AR (**C–E**); tumour cells show diffuse expression of the neuroendocrine marker chromogranin (**F**). [Colour figure can be viewed at wileyonlinelibrary.com]

In a representative case of primary de novo small cell carcinoma of the prostate, the initial prostate core biopsy revealed mixed prostatic adenocarcinoma and small cell carcinoma (Figure 3). FOXA1 was expressed in both components, whereas NKX3.1, PSMA and AR were expressed only in the adenocarcinoma component and lost in the small cell carcinoma component. The small cell carcinoma component showed diffuse expression of neuroendocrine markers (chromogranin, synaptophysin) with a high Ki67 index. Another example was a lymph node biopsy with metastatic small cell carcinoma of the prostate (Figure 4). In this case, FOXA1 was strongly expressed in the tumour cells, which were diffusely positive for neuroendocrine markers and negative for NKX3.1, PSMA and AR.

**Figure 5:**
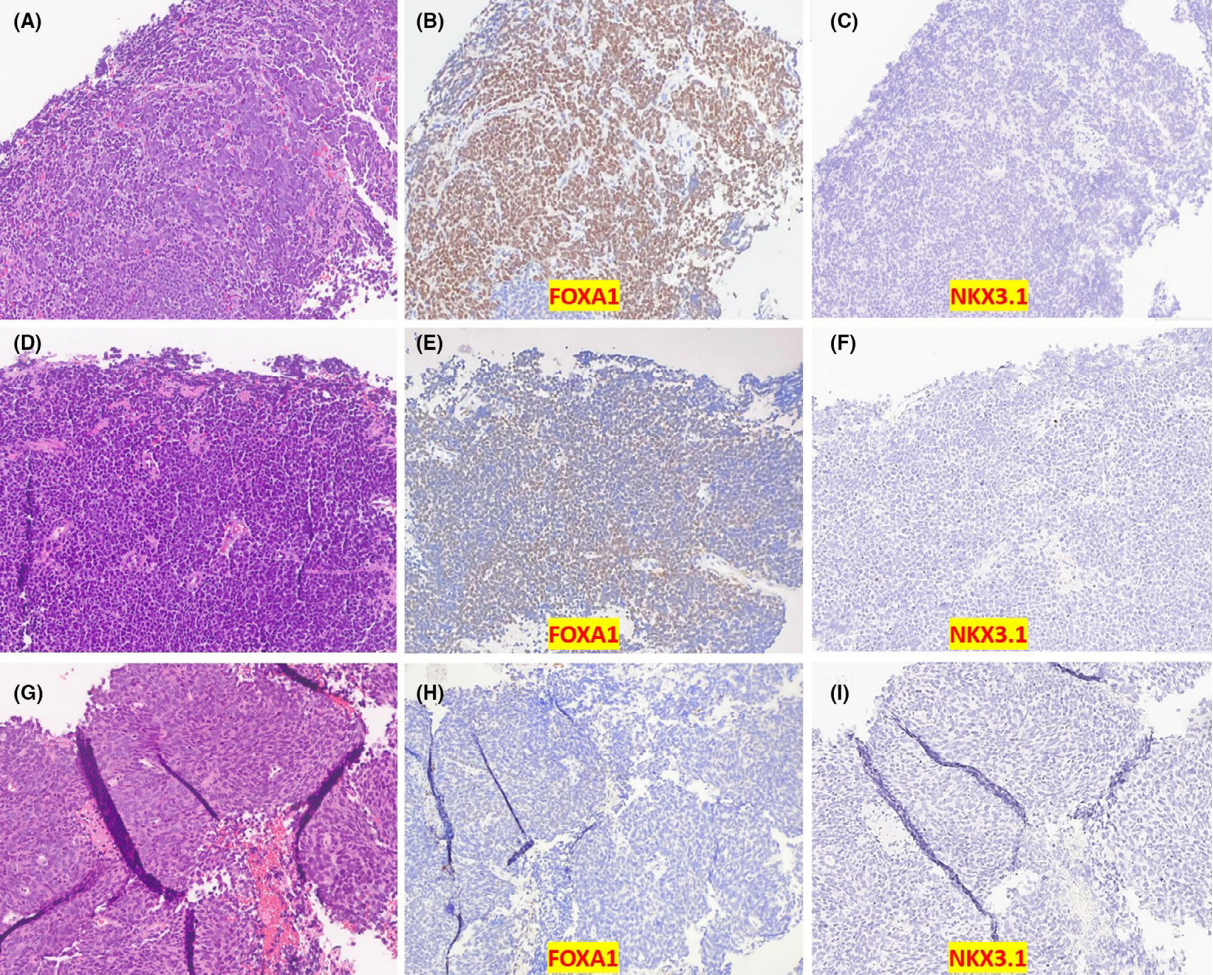
Different FOXA1 expression patterns in primary/metastatic prostatic small cell carcinoma. (**A–C**) High positivity of FOXA1 staining in tumour cells; (**D–F**) Intermediate positivity of FOXA1 staining in tumour cells; (**G–I**) Negative FOXA1 staining in tumour cells. By contrast, NKX3.1 staining is negative in all three cases. [Colour figure can be viewed at wileyonlinelibrary.com]

FOXA1 expression in small cell carcinoma of the prostate shows a variable spectrum from diffuse strong (high positivity), focal strong/patchy weak (intermediate positivity), to focal weak (low positivity) (Figure 5). Among 10 primary prostatic small cell carcinomas, 4 (40%) cases showed high positivity of FOXA1 staining, 2 (20%) were intermediate and 2 (20%) were low. Among 21 metastatic cases, 3 (14%) cases showed high positivity of FOXA1 staining, 4 (19%) were intermediate and 5 (24%) were low.

### FOXA1 expression in other tumour types

**Figure 6:**
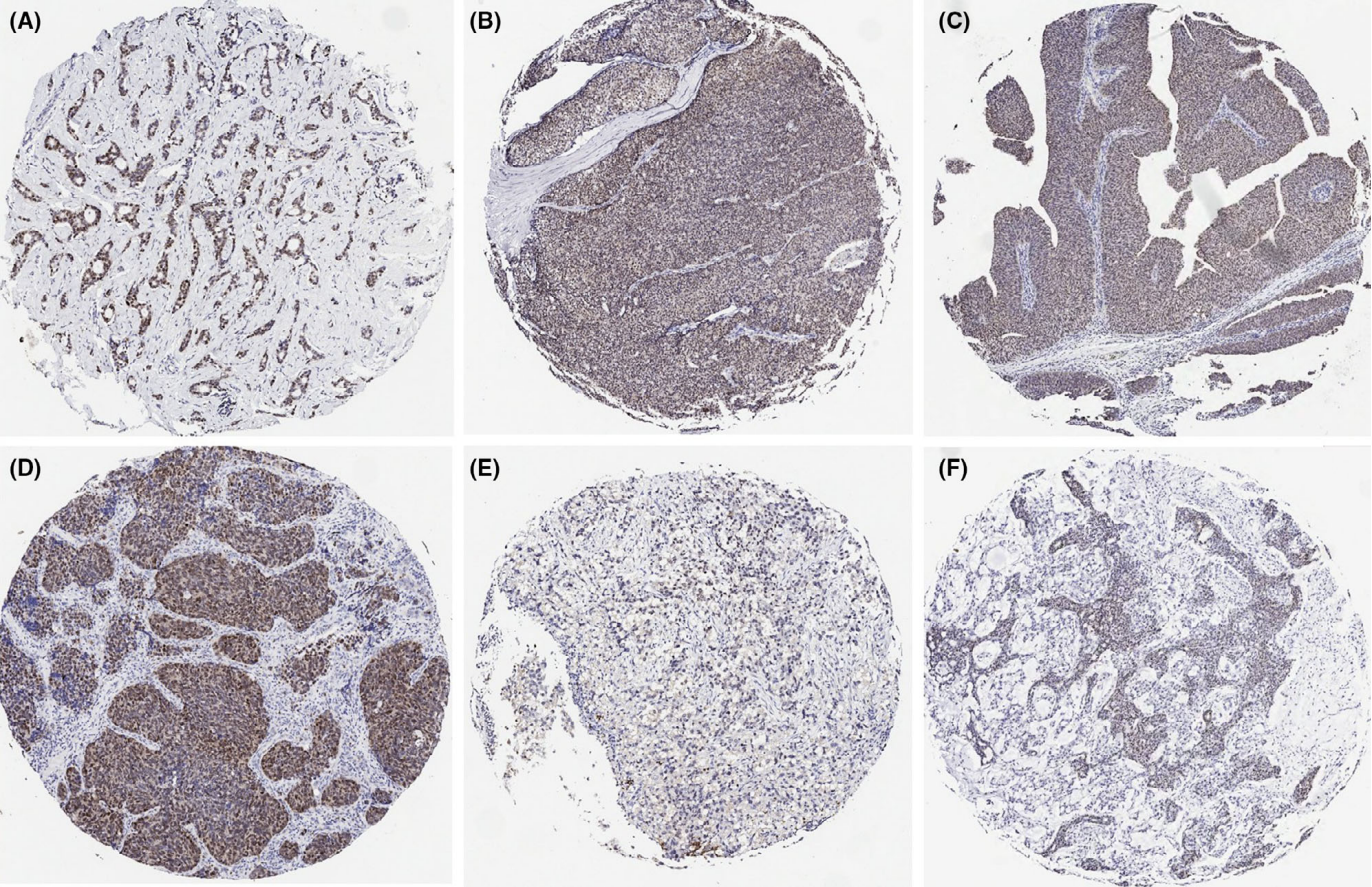
FOXA1 expression in other tumours based on tissue microarray. Intermediate/high positivity of FOXA1 staining in (**A**) breast invasive carcinoma, (**B**) breast neuroendocrine carcinoma, (**C**) urothelial carcinoma of bladder, (**D**) lung squamous cell carcinoma and (**E**) low positivity of FOXA1 staining in (**E**) lung adenocarcinoma and (**F**) testicular yolk sac tumour. [Colour figure can be viewed at wileyonlinelibrary.com]

Utilizing tissue microarrays, we evaluated FOXA1 expression across multiple tumour types (Table 2). FOXA1 was frequently expressed in 25 (57%, from low to high positivity) of 44 invasive breast carcinomas and 15 (22%, from low to high positivity) of 68 urothelial carcinomas of bladder/kidney (Figure 6). In addition, FOXA1 was detected in 2 (6%, low positivity) of 31 lung adenocarcinomas, 1 (3%, high positivity) of 30 lung squamous cell carcinomas, 2 (2%, low positivity) of 125 colon adenocarcinomas and 1 (10%, low positivity) of 10 testicular yolk sac tumours. No FOXA1 expression was detected in 47 renal cell carcinomas, 64 thyroid tumours, 96 hepatocellular carcinomas or other 48 testicular germ cell tumours.

**Table 2:** FOXA1 expression in tumours of different organs

FOXA1 expression		Negative	Positive			Total
			Low	Intermediate	High	
Breast	Invasive mammary carcinoma	19 (43%)	10 (23%)	11 (25%)	4 (9%)	44 (100%)
Bladder	Low‐grade urothelial carcinoma	4 (67%)	0	2 (33%)	0	6 (100%)
	High‐grade urothelial carcinoma	39 (75%)	3 (6%)	7 (13%)	3 (6%)	52 (100%)
Kidney	Clear cell renal cell carcinoma	33 (100%)	0	0	0	33 (100%)
	Papillary renal cell carcinoma	9 (100%)	0	0	0	9 (100%)
	Chromophobe renal cell carcinoma	5 (100%)	0	0	0	5 (100%)
	Invasive urothelial carcinoma	10 (100%)	0	0	0	10 (100%)
	Angiomyolipoma	2 (100%)	0	0	0	2 (100%)
Thyroid	Papillary thyroid carcinoma	26 (100%)	0	0	0	26 (100%)
	Thyroid adenoma	25 (100%)	0	0	0	25 (100%)
	Follicular thyroid carcinoma	7 (100%)	0	0	0	7 (100%)
	Medullary thyroid carcinoma	6 (100%)	0	0	0	6 (100%)
Lung	Adenocarcinoma	29 (94%)	2 (6%)	0	0	31 (100%)
	Squamous cell carcinoma	29 (97%)	0	0	1 (3%)	30 (100%)
Colon	Adenocarcinoma	123 (98%)	2 (2%)	0	0	125 (100%)
Liver	Hepatocellular carcinoma	96 (100%)	0	0	0	96 (100%)
Testis	Seminoma	26 (100%)	0	0	0	26 (100%)
	Embryonal carcinoma	12 (100%)	0	0	0	12 (100%)
	Teratoma	10 (100%)	0	0	0	10 (100%)
	Yolk sac tumour	9 (90%)	1 (10%)	0	0	10 (100%)

### FOXA1 expression in neuroendocrine tumours/carcinomas of various origins

We also examined FOXA1 expression in well‐differentiated neuroendocrine tumours and neuroendocrine carcinomas of various organs (Table 3). In our limited cohort, only 2 of 4 breast neuroendocrine carcinomas showed low to intermediate FOXA1 expression (Figure 6). In contrast, no FOXA1 expression was observed in neuroendocrine tumours/carcinomas from the bladder (*n* = 5), lung (*n* = 47), gastrointestinal tract (*n* = 30), pancreas (*n* = 6), gynaecological tract (*n* = 9) or Merkel cell carcinoma of the skin (*n* = 5).

**Table 3:** FOXA1 expression in neuroendocrine tumour/carcinoma of different organs

FOXA1 expression		Negative	Positive			Total
			Low	Intermediate	High	
Breast	Neuroendocrine carcinoma	2 (50%)	1 (25%)	1 (25%)	0	4 (100%)
Bladder	Neuroendocrine carcinoma	5 (100%)	0	0	0	5 (100%)
Lung	Neuroendocrine tumour	3 (100%)	0	0	0	3 (100%)
	Small cell carcinoma	44 (100%)	0	0	0	44 (100%)
Gastrointestinal tract	Neuroendocrine tumour	15 (100%)	0	0	0	15 (100%)
	Neuroendocrine carcinoma	15 (100%)	0	0	0	15 (100%)
Pancreas	Neuroendocrine tumour	3 (100%)	0	0	0	3 (100%)
	Neuroendocrine carcinoma	3 (100%)	0	0	0	3 (100%)
Gynaecological tract	Neuroendocrine carcinoma	9 (100%)	0	0	0	9 (100%)
Skin	Merkel carcinoma	5 (100%)	0	0	0	5 (100%)

## Discussion

As a pioneer transcription factor, FOXA1 plays a crucial role in prostate development and prostate cancer biology.^2^, ^6^, ^7^ In normal prostate tissue, FOXA1 facilitates AR binding to chromatin, thereby regulating the transcription of AR target genes involved in cell growth and differentiation. FOXA1 is also implicated in the molecular pathogenesis of prostate cancer. Recurrent mutations and altered expression of FOXA1 have been reported in 9% of primary prostate cancers, 13% of metastatic castration‐resistant prostate cancers and 25% of neuroendocrine prostate cancers.^8^, ^9^ These alterations may influence tumour behaviour by reprogramming AR signalling and promoting lineage plasticity.^10^ Several studies have shown that *FOXA1*‐mutated prostate cancers are associated with poorer clinical outcomes compared to wild‐type cases.^2^, ^11^, ^12^ Moreover, FOXA1 dysregulation has been linked to the emergence of aggressive variants, including neuroendocrine prostate cancer. Kim *et al*. demonstrated that FOXA1 loss via shRNA induced a neuroendocrine phenotype in prostate cancer cells, and suggested that FOXA1 may act as a suppressor of androgen‐independent neuroendocrine transformation.^13^

In our study, FOXA1 showed a nuclear staining pattern and high sensitivity similar to NKX3.1 in normal prostatic glands as well as in both primary and metastatic prostatic adenocarcinomas. Remarkably, FOXA1 was detected in 8 of 10 (80%) primary and 12 of 21 (57%) metastatic small cell carcinomas of the prostate, despite the complete loss of traditional markers such as NKX3.1, PSA, PSMA and AR in these cases. These findings suggest that loss or reduction of FOXA1 expression may contribute to transformation from adenocarcinoma to small cell carcinoma and subsequent progression in a subset of cases. However, FOXA1 loss is clearly not essential in all cases, indicating that other transcriptional regulators or molecular pathways are likely involved in this lineage transformation. Clinically, prostatic neuroendocrine carcinoma commonly loses expression of conventional prostatic markers, including NKX3.1. This loss frequently poses a significant diagnostic challenge for surgical pathologists when evaluating metastatic neuroendocrine tumours in patients with a prior history of prostate cancer, particularly in distinguishing a prostatic origin from a new primary malignancy. In this clinical setting, our findings suggest that positive FOXA1 expression in the tumour would support a prostatic origin and may help resolve this diagnostic dilemma.

Interestingly, Park *et al*. demonstrated that FOXA2, another FOXA family member, was strongly expressed in 15 of 20 (75%) cases of prostatic small cell carcinoma, but in only 9 of 215 (4.2%) prostatic adenocarcinomas.^14^ Their study proposed FOXA2 as a sensitive and specific marker for small cell carcinoma of the prostate, but not for prostatic adenocarcinoma. However, FOXA2 has been reported to be expressed in all types of lung neuroendocrine tumours and rarely in lung adenocarcinoma.^15^ In contrast, in our microarray analysis of 106 well‐differentiated neuroendocrine tumours and neuroendocrine carcinomas across various organs, only 2 of 4 breast neuroendocrine carcinomas showed FOXA1 expression. This suggests that FOXA1 may have higher specificity than FOXA2 in diagnosing small cell carcinoma of prostatic origin. Together, our findings and those of others highlight potentially divergent roles for FOXA1 and FOXA2 in prostate cancer tumorigenesis, including small cell transformation. While both FOXA1 and FOXA2 have been implicated in prostate cancer development, lineage plasticity and treatment resistance,^6^, ^10^, ^16^, ^17^ whether they act synergistically or antagonistically remains to be elucidated.^2^

Using tissue microarrays, we further evaluated FOXA1 expression in other tumour types, with immunoexpression identified in 25 of 44 (57%) breast cancers, 15 of 68 (22%) of bladder/kidney urothelial carcinomas, 3 of 61 (5%) lung carcinomas, 2 (2%) of 125 colon adenocarcinomas and 1 of 10 (10%) testicular yolk sac tumours. These results are consistent with prior literature, in which expression of FOXA1 has been reported in breast, oesophagus, bladder, colon and lung cancers.^2^, ^18^, ^19^, ^20^, ^21^, ^22^ These data suggest that FOXA1 is less specific than NKX3.1 as a prostatic marker. We noticed different frequencies of expression between our study and some published reports. One possible cause is that our study used a commercial tissue microarray, whereas other studies used whole‐slide tissue sections or fresh institutionally constructed tissue microarrays with potentially improved tissue quantity and quality. In addition, the FOXA1 antibody and IHC conditions we used may be different from those in other studies, leading to differential sensitivity for detecting FOXA1 expression.

In summary, we have shown that FOXA1 can serve as a valuable adjunct IHC marker to confirm prostatic origin in diagnostically challenging cases, especially in small cell carcinoma of the prostate that loses traditional prostatic marker expression. This unique expression profile also makes FOXA1 a potential candidate for future targeted therapy and prognostic stratification in prostate cancer, including small cell carcinoma. Given the relatively small size of our cohort, these findings should be validated in independent studies. Future work is needed to study the expression of FOXA1 in other aggressive subtypes of prostate cancer and to elucidate the molecular mechanisms by which FOXA1 influences lineage progression and therapeutic resistance in prostate cancer.

## Conflict of interest statement

The authors declare that they have no conflict of interest.

## Data Availability

The data that support the findings of this study are available from the corresponding author upon reasonable request.
